# CTSA pharmacies: Contribution to research and public health during the COVID-19 pandemic

**DOI:** 10.1017/cts.2021.13

**Published:** 2021-02-18

**Authors:** Robert B. MacArthur, Ohad S. Bentur, Ian C. MacArthur, Anna S. Bartoo, Donna L. Capozzi, Jason A. Christensen, Amber L. Johnson, Kuldip Patel, Barry S. Coller

**Affiliations:** 1 Rockefeller University Hospital Pharmacy, The Rockefeller University, New York, NY, USA; 2 Allen and Frances Adler Laboratory of Blood and Vascular Biology, The Rockefeller University, New York, NY, USA; 3 Medical Scientist Training Program, Albert Einstein College of Medicine, Bronx, NY, USA; 4 Pharmacy Department, Mayo Clinic, Rochester, MN, USA; 5 Ambulatory Oncology Pharmacy Services, Hospital of the University of Pennsylvania Pharmacy Department, Philadelphia, PA, USA; 6 Pharmacy Department, Duke University Hospital, Durham, NC, USA

**Keywords:** Pharmacy, COVID-19, drug shortage, vaccination, investigational drugs, supply and distribution, teleworking, biomedical research

## Abstract

**Introduction:**

In March 2020, academic medical center (AMC) pharmacies were compelled to implement practice changes in response to the COVID-19 pandemic. These changes were described by survey data collected by the Clinical and Translational Science Awards (CTSA) program which were interpreted by a multi-institutional team of AMC pharmacists and physician investigators.

**Methods:**

The CTSA program surveyed 60 AMC pharmacy departments. The survey included event timing, impact on pharmacy services, and corrective actions taken.

**Results:**

Almost all departments (98.4%) reported at least one disruption. Shortages of personal protective equipment (PPE) were common (91.5%) as were drug shortages (66.0%). To manage drug shortages, drug prioritization protocols were utilized, new drug supply vendors were identified (79.3%), and onsite compounding was initiated. PPE shortages were managed by incorporating the risk mitigation strategies recommended by FDA and others. Research pharmacists supported new clinical research initiatives at most institutions (84.0%), introduced use of virtual site visits, and shipped investigational drugs directly to patients. Some pharmacies formulated novel investigational products for clinical trial use. Those AMC pharmacies within networked health systems assisted partner rural and inner-city hospitals by sourcing commercial and investigational drugs to alleviate local disease outbreaks and shortages in underserved populations. Pharmacy-based vaccination practice was expanded to include a wider range of pediatric and adult vaccines.

**Conclusion:**

The COVID-19 pandemic radically altered hospital pharmacy practice. By adopting innovative methods and adapting to regulatory imperatives, pharmacies at CTSA sites played an extremely important role supporting continuity of care and collaborating on critical clinical research initiatives.

## Introduction

Beginning in March 2020, numerous hospitals were jolted by the COVID-19 pandemic. Intensive care unit beds were filling; drug and personal protective equipment (PPE) supplies dwindled in New York, New Jersey, Connecticut, and Massachusetts; universal social distancing was imposed; and new COVID-19 research initiatives became a priority.

Pharmacists were among the first responders who assisted in pivoting hospital and research services to meet this unforeseen demand. Many of these changes were captured via a survey distributed by the Clinical and Translational Science Awards (CTSA) Program which collected responses from 60 CTSA institutional pharmacies.

Institutional pharmacies managed through drug and PPE shortages via modifications of in-house prescribing practices and expansion of approved drug and PPE suppliers. Hospital pharmacists learned to work remotely. Some pharmacy-based clinical services such as coagulation and vaccination clinics altered practice to better and more safely accommodate patient needs. Tertiary hospitals in networks assisted partner rural and inner-city hospitals by supplying commercial and investigational drug products, thereby addressing the needs of underserved racial and ethnic minority populations.

Research pharmacy operations were expanded by bringing on new staff, expanding operating hours, modifying drug distribution methods, switching to virtual monitoring visits (replacing onsite sponsor and regulatory agency visits), and providing certain services remotely. In some cases, research pharmacists participated on drug development teams and formulated investigational drug products.

This paper describes COVID-19-related changes to institutional and research pharmacy practice and discusses the lessons learned that are anticipated to carry forward, beyond the pandemic.

## Methods

In August 2020, the CTSA organized writing teams and provided them with support via the Center for Leading Innovation and Collaboration (CLIC). A “Pharmacy: Procurement, Compounding, Formulation” writing team was formed, composed of pharmacists and physician scientists from multiple CTSA academic medical centers (AMCs). The group assisted with the development of the survey, which was then organized, programmed, and forwarded to 60 CTSA sites. Following review by the writing team, survey responses were interpreted and described by comparing pre-COVID-19 practices to modified practices. Not every pharmacy responded to every question, and results are presented as the number of affirmative responses/number responding to that survey question, and by percent, calculated based upon the numerical result. The survey is included as a supplemental table in the companion paper entitled ‘Re-engineering The Clinical Research Enterprise in Response to COVID-19: The Clinical Translational Science Award (CTSA) Experience and Proposed Playbook for Future Pandemics.’

## Results

### Pre-COVID-19 Practices

Proper hospital pharmacy practices are vital to safe and effective patient therapy and thus require standardization, documentation, and verification. As such, hospital pharmacies are highly regulated by legislation, regulations, and policies at the federal, state, and local levels. In addition, hospital pharmacy policies and procedures, as well as staff training and monitoring, need to be fully integrated with those of the medical and nursing departments. All of these constraints make changes to pharmacy practice a slow and deliberate process. Below are examples of procedures that were well established before the COVID-19 pandemic and thus were difficult to modify.

Before COVID-19, hospital investigational drug services (IDS) routinely relied upon the study sponsor or Investigational New Drug (IND) Application holder to provide all investigational drug products and general drug dispensing procedures. As CTSA-affiliated hospitals are very actively involved with ongoing clinical research, IDS services are staffed by IDS-dedicated pharmacists and technicians who almost always work within a hospital pharmacy department. The number of IDS staff is often limited, so IDS services are available to Investigators during common business hours, and off hour and weekend coverage either is available by on-call staff (which introduces a time delay) or not at all.

Prior to COVID-19, the study startup process was performed exclusively onsite, within the hospital IDS pharmacy area. Inventory and dispensing tasks were performed via computer data entry using validated software. Data capture for other essential tasks relied on manual data entry onto hard copy paper records, to capture shipping, receiving, enrollment, randomization, calculations, patient accountability, distribution, and other activities. This manual approach is required per Good Documentation Practices (GDPs), and these activities must be captured per Good Clinical Practice (GCPs) standards [1,2]. The data capture process must happen in real time and therefore documents are commonly organized within study-specific binders within easy reach to IDS staff. Among other uses, these records document investigational drug chain of custody and are complementary to the hospital electronic health record (EHR) which documents the majority of other patient care activities.

Also pre-COVID-19, IDS drug distribution was limited to hospital inpatient and outpatient clinics, meaning that only under emergency circumstances with prior sponsor approval were hospital pharmacies permitted to ship investigational medications directly to a patient’s home. In addition, the sponsor routinely performs multiple onsite study audits of the IDS. These practices are well described in the American Society of Health-System Pharmacists (ASHP) statement on Investigational Pharmacy Practice [3].

Hospital pharmacy commercial drug supply chains, although continuously stressed by drug shortages for the past 10 or more years, have been able to meet institutional needs by varying procurement strategies. In many institutions, PPE supply was managed by hospital central supply services.

Pharmacy-based clinical services, including vaccine administration programs, anticoagulation clinics, and diagnostic testing programs, have been limited by state and federal regulations, as well as by institutional acceptance. Many of these programs have thrived and proven their value within communities, where they address unmet medical needs of diverse, underserved populations. Despite that, program expansion has been stifled by regulations.

### Modified COVID-19 Practices

**Fig. 1.**
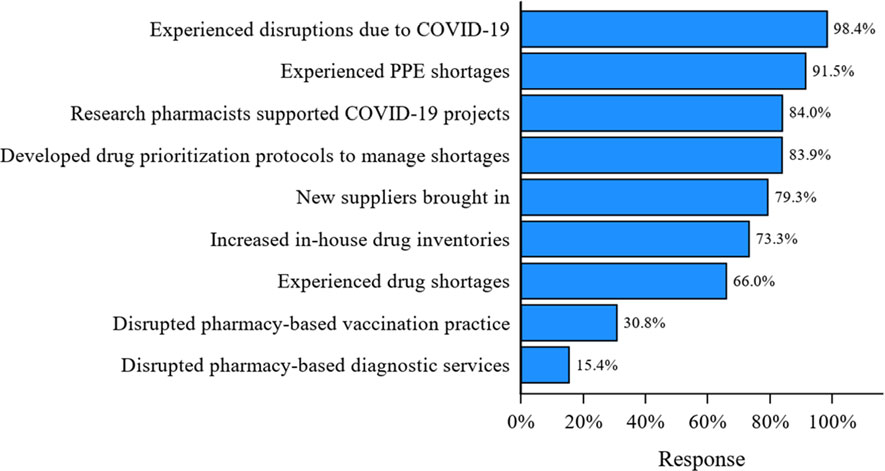
Clinical and Translational Science Awards (CTSA) Pharmacy Survey Results. PPE, personal protective equipment.

Figure 1 depicts some of the modifications made to pharmacy practice in response to the COVID-19 pandemic by CTSA institutions by the time the survey was distributed to Hubs in October 2020.

### Hospital Pharmacy

Almost all CTSA AMC pharmacies reported some type of COVID-19 pandemic-related disruption (59/60, 98.4%) that began either in January to March 2020 (49/60, 81.7%) or April to June (10/60, 16.7%).

All responders noted that the COVID-19 pandemic modified IDS services (52/52, 100%). COVID-19-related pharmacy PPE (43/47, 91.5%) and drug shortages (31/47, 66.0%) were very common. Hospital pharmacy-based vaccination practices (16/52, 30.8 %,) and diagnostic services (8/52, 15.4%) were modified at some institutions.

### Research Pharmacy

The majority of CTSA institutions rely upon their research pharmacists to support COVID-19 research projects (42/50, 84.0%). During the pandemic, it was essential that pharmacists worked in real time with clinical departments, especially Infectious Diseases, Cardiology, Neurology, and Critical Care, in addition to the Institutional Review Board (IRB) and clinical research investigators.

A small number of pharmacies reported that a research pharmacist participated in a product development research team. Some of these activities included commercial drug repurposing studies (21/49, 42.9%). Some pharmacies sourced active pharmaceutical ingredient(s) (API) and formulated novel products for use in COVID-19 clinical trials (4/44, 9.1%). Sourcing of API involves finding a commercial source of pure active ingredients that are produced in compliance with Good Manufacturing Practices and ideally have an active Drug Master File registered at FDA. Formulating novel products involves compounding API into formulations suitable for human use, either under an IND or in response to a prescription for a specific patient.

As an example of a rapidly initiated API and formulation project, a CTSA hospital pharmacy formulated an investigational inhaled aerosol product (aerosolized hydroxychloroquine sulfate). The project began in late March 2020, and the formulation was developed the following month and placed into initial stability testing by early May 2020. In parallel, the Investigator and study team compiled the necessary information, including existing preclinical toxicology and early human studies, into an IND application that was filed under an Emergency Use Authorization (EUA) in mid-May. The EUA allowed the Food and Drug Administration (FDA) to work closely with the Investigators, and IND-related correspondence was exchanged with the FDA once or twice weekly. With that level of attention, the IND was allowed to proceed by early June. IRB approval was received by mid-June, and the first subjects were enrolled into the Phase 1 trial during the last week of June 2020. Thus, the time from project inception to first subject dosing was approximately 3.0 months. All of the importation of the API, formulation development work, contracting with formulation testing laboratories, and sterile compounding was performed by the CTSA hospital pharmacy working closely with the physician-investigators.

Other products compounded by multiple CTSA AMC pharmacies in response to shortages include N95 fit test solutions; COVID-19 viral transport medium tubes, hand sanitizer, and chlorine cleaning solutions; and active and placebo products for early phase repurposing trials.

Additional changes to research pharmacy practice included shipping investigational drug products directly to patients at home. Note that IND sponsors and IRBs issued memos at the beginning of the pandemic approving this practice, so these new activities were not considered protocol violations and no corrective and prevention actions (CAPAs) were required. It became obvious that such shipments were essential to maintaining protocol-directed treatments for patients in the setting of quarantine and travel restrictions.

### Drug Shortages

Drug shortages have been a hallmark of the COVID-19 pandemic and were reported by most CTSA pharmacies surveyed (31/47, 66.0%). There was a sudden rapid expansion in the use of medications such as albuterol, azithromycin, cisatracurium, dexamethasone, dexmedetomidine, epinephrine, fentanyl, hydromorphone, hydroxychloroquine sulfate, midazolam, norepinephrine, phenylephrine, propofol, rocuronium, tocilizumab, and vasopressin [4]. Some investigational agents, such as remdesivir, were in short supply and required allocation protocols and “lotteries.” Management of these shortages required pharmacies to know the available inventory of these products day-to-day and to make predictions about future use when ordering, which is a labor-intensive task that requires ongoing vigilance.

Many drug shortages were managed via institutional treatment guidelines (also referred to as Drug Prioritization Protocols), which directed the judicial use of drugs and drug classes utilized to treat COVID-19-related complications, such as anticoagulants, neuromuscular blocking agents, and benzodiazepines, and prioritizing use of those products that were available within existing supply chains at a given time. The guidelines often recommended alternative drug treatments if the more commonly used products were not available. At most CTSA AMC institutions, these treatment guidelines are developed within the Pharmacy and Therapeutics Committee (P&T) by subcommittees comprised of subject matter experts, including physicians, pharmacists, nurses, and pharmacologists. Once approved by the P&T, the recommendations are distributed via formal memos to the relevant medical departments for implementation and are also programmed into the EHR. Institutional Pharmacies also managed drug shortages by finding new suppliers and distributors of drug products (23/29, 79.3%) and pre-emptive drug stockpiling (22/30, 73.3%).

### PPE Shortages

As mentioned, PPE shortages were reported by 91.5% of respondents. An extensive number of mitigation strategies were developed by the FDA and other regulatory and state agencies, a number of which are summarized in Table 1.

**Table 1.** Food and Drug Administration (FDA) and United States Pharmacopeia (USP) guidance on managing COVID-19-related personal protective equipment (PPE) shortages

Food and Drug Administration
Questions about Personal Protective Equipment (03/11/2020) [5]
Surgical Mask and Gown Conservation Strategies - Letter to Health Care Providers (4/27/2020) [6]
Temporary Policy Regarding Nonstandard PPE Practices for Sterile Compounding by Pharmacy Compounders not Registered as Outsourcing Facilities During the COVID-19 Public Health Emergency (5/14/2020) [7]
Temporary Policy for Compounding of Certain Drugs for Hospitalized Patients by Outsourcing Facilities During the COVID-19 Public Health Emergency (5/21/2020) [8]
FAQs on Shortages of Surgical Masks and Gowns During the COVID-19 Pandemic (06/19/2020) [9]
Coronavirus (COVID-19) and Medical Devices (12/10/2020) [10]
United States Pharmacopeia
USP Response to Shortages of Garb and Personal Protective Equipment for Sterile Compounding During COVID-19 Pandemic (3/18/2020) [11]
Operational Considerations for Sterile Compounding During COVID-19 Pandemic (12/7/ 2020) [12]

Many CTSA pharmacies reported that PPE is distributed within their institutions from central locations and so is not managed by the pharmacy. Thus, institution-wide policies were required to address the shortages. The PPE used by hospital pharmacies (e.g., sterile hooded coveralls, nonsterile hazardous drug protective gowns, sterile gloves) differs from that used in patient care areas (e.g., isolation gowns, face shields, non-sterile gloves), making the pharmacy supply issues unique.

Pharmacies worked with central supply departments to identify new PPE sources. Also PPE supplies were donated by both international organizations and “well provisioned” healthcare institutions to those hospitals in urgent need [13,14].

### Vaccination and Diagnostic Practice

As the COVID-19 pandemic evolved, the US Department of Health and Human Services (HHS) via the Public Readiness and Emergency Preparedness Act (PREP Act) removed some of the restrictions on pharmacy-based vaccination programs in order to improve public vaccination compliance rates in the setting of lock-downs, closed physician offices, and social distancing.

In an April 8, 2020, guidance HHS noted that the vast majority of Americans live close to a retail or independent community-based pharmacy. As of 2018, 90% of Americans live within 5 miles of a community pharmacy [15]. That proximity reduces travel to testing locations, which is an important mitigation measure. Pharmacists also have strong relationships with local medical providers and nearby hospitals to appropriately refer patients when necessary [15]. Via this guidance, HHS authorized licensed pharmacists to order and administer FDA-approved COVID-19 diagnostic tests, including serology tests. Under the Act pharmacists are “covered persons,” shielding them from liability. Further, on September 3, 2020, another PREP guidance was issued that authorized pharmacists, again as covered entities, to order and administer FDA-approved COVID-19 vaccines to persons ages three or older under a set of very specific guidelines [16]. Also under PREP, on October 20, 2020, trained pharmacy technicians and interns, when acting under the supervision of a qualified pharmacist, were also allowed to administer COVID-19 vaccines to persons ages three or older. Additionally, trained pharmacy technicians and interns were also permitted to administer other vaccines, namely, those vaccines recommended by the Advisory Committee on Immunization Practices (ACIP) as outlined by the ACIP standard immunization schedule [17].

While much of this practice expansion applied to retail pharmacies, some CTSA pharmacies reported changes in vaccination practices (12/14, 85.7%) and diagnostic test practices (3/6, 50.0%), such as performing prothrombin time International Normalized Ratio (INR) tests for anticoagulation clinics.

At one CTSA hospital, the pharmacy participated in emergency and disaster response with pop-up vaccination tents placed in parking lots. At another hospital, the pharmacy’s anticoagulation clinic, for the first time, permitted “drive-up” point-of-care INR testing, allowing collection of patient samples outside of the building. Some anticoagulation clinic visits were transitioned to telehealth consultations.

For hospital employee and family-based programs, pharmacy vaccination services were expanded at some institutions. Two CTSA pharmacies reported that Influenza Vaccination Clinics opened at the routine time of the year (i.e., early Fall 2020) but then closed earlier than in past years, due to staffing limitations, social distancing requirements, and tight influenza vaccine supplies. Hospital employees were encouraged to get vaccinated early, and those unable to attend the hospital clinic were directed to local community pharmacies or vaccination clinics.

## Lessons Learned

Practices implemented during the pandemic which may endure are listed in Table 2:

**Table 2.** Lessons learned and possible long-term changes to standard practice

**Pharmacists Working Remotely** - Hospital pharmacists performing remote patient drug order entry and verification, writing policies, standard operating procedures, and performing medication counseling via web platforms. - Research pharmacists writing study specific procedures, ordering equipment and supplies, writing batch records, performing remote patient drug order entry and verification. - 100% virtual site visits, by sponsors, state, and federal agencies. - Many state regulations were temporarily modified to permit pharmacists to work remotely. **Broadened Research Pharmacy Practice** - Shipping investigational drugs for oral and topical use directly to study patients. - Collaborations between institutions to manage scarce research drugs supplies. - Consolidating responsibilities among institutions that require around the clock availability such as patient protocol entry and randomization. - Supporting home infusion of investigational drugs. - Trending toward decentralized clinical trial structures. **Addressing Shortages and Inequities** - Allocation protocols accommodate fluctuating drug supplies and treatment alternatives. - Institutional Drug Prioritization Protocols are integrated into electronic health records to guide order entry. - Sequestered per patient drug supplies to ensure a treated patient can complete a full treatment course, before a second patient is enrolled, under both Investigational New Drug (INDs) and Emergency Use Authorizations (EUAs). - Tertiary institutions contract with multiple drug wholesalers, pharmaceutical companies, and 503B suppliers to support network partner rural and underserved hospitals. **Expanded Vaccination and Clinical Practice** - Outreach to the public via Drive-Thru and Pop-Up clinics includes both vaccination and diagnostic testing ordered by and administered by pharmacists and pharmacy technicians. - Expansion into pediatric vaccination via amendment of the Public Readiness and Emergency Preparedness (PREP) Act. - Pharmacy services offered via telehealth platforms, in addition to in on-site clinics.

### Pharmacists Working Remotely

Prior to the pandemic, opportunities for hospital pharmacists to work from home were very limited, in many cases due to state regulations [18]. However, given the risk of exposure to COVID-19 on public transportation, in elevators, and within hospital, coupled with the risk of contagion while working in small pharmacy spaces, some pharmacists were now required to work at home. This was in response to the concern that if many hospital pharmacy staff became ill, hospital operations and patient care might be disrupted. Based on this concern, many state pharmacy boards temporarily revoked regulations that prevented pharmacists from working remotely.

This forced a rethinking of hospital pharmacist and technician workflow. Methods were developed to securely share departmental documents while offsite and to securely connect to hospital pharmacy patient records and drug order entry systems from remote locations. In many cases, this relied upon using a combination of secure, shared, cloud-based encrypted repositories (e.g., Box, DropBox, One Drive, and others), together with remote secure encrypted personal computer access.

With these changes, and others, pharmacists learned to perform basic job functions remotely, such as patient drug order entry and verification, and drug and supply ordering (Table 2). Medication counseling via secure video platforms became another newly learned skill. The new workflow still required a small number of pharmacists and technicians to be onsite to perform the daily manual tasks of inventory maintenance, dispensing, and compounding, among other essential functions.

The sudden increased reliance upon “remote pharmacists” adds to the more than 10 years of experience gained with remote pharmacy practice in other settings. Up until recently, use of remote pharmacists and technicians was limited primarily to rural areas, nursing homes, long term care facilities, and some midsize hospitals where remote pharmacists provide off-hour pharmacy services when demand for pharmacy services is lower [19]. Much of this is driven by both a limited local pharmacist and technician pool in rural regions, and the economics of paying pharmacists as hourly contractors rather than as full-time employees.

The sudden successful mass implementation of pharmacists working remotely will likely continue to expand due to economic incentives, employer interest, patient needs, and more receptive state pharmacy boards. Whether or not states will eventually return to prepandemic restrictions on remote working remains to be seen.

### Broadened Research Pharmacy Practice

Starting as early as March 2020, research pharmacy staff embraced remote working and virtual monitoring, as did study sponsors, federal, and state agencies. As a result, many IDS groups are closer to becoming “paper-free” as sponsor remote monitoring visits now include the secure exchange of electronic records and images, with an occasional follow-up video interview with the IDS pharmacist or technician to answer any remaining questions. Some IDS pharmacies have created virtual project startup videos to further automate and simplify the sponsor site-introduction and monitoring process. Combined, these new practices create efficiencies in time, scheduling, and reduction of travel for all involved.

Research pharmacists working remotely likely will also continue. While the need to have staff onsite to perform dispensing will not change, a number of tasks are now performed remotely, including drafting study-specific procedures, ordering equipment and supplies, and writing standard operating procedures, batch records, and other documents. Also, the study initiation process, which includes creating EHR drug profiles, computerized physician order entry (CPOE) screens, and educating pharmacy and hospital staff, can be performed remotely.

Shipping investigational drugs directly to patients at home or in long-term health facilities, and coordinating shipments with study sponsors and home health agencies, has now become a common practice, and so may continue. Notably, this practice was permitted by the National Cancer Institute as early as March 13, 2020 [20,21], and around the same time by many clinical trial sponsors to ensure patient safety and continuity of care while under research protocols.

This new practice aligns with a more generalized trend towards decentralized clinical trials, in that the research pharmacies can, if permitted, simplify study participation for patients and caregivers. Research pharmacies can provide both patient-specific dispensing and investigational drug shipment services. This path is similar to that already followed by the growing “Specialty Pharmacy” industry where some FDA-designated 503A pharmacies (e.g., mail order pharmacies, compounding pharmacies, closed door pharmacies, patient direct pharmacies) interact directly with patients and providers to dispense medications by courier and the US Postal Service. Research pharmacies could further expand their existing services to include management of at-home dispensing machines like “Spenser”^®^[22], along with online patient diaries, and video dosing.

### Addressing Shortages

As drug shortages are expected to continue, so will the use of institutional drug prioritization protocols that conserve and allocate scarce medications.

These institutional protocols encourage the selection of alternative drug products, in the setting of drug product shortages, by navigating users (physicians, licensed institutional practitioners, physician assistants, and others) toward available medications and away from products in short supply. These protocols have been in use for over 40 years, although never on a nation-wide basis during a global pandemic.

Hospitals must continue to maintain a robust group of drug wholesalers and distributors, which now typically also includes both FDA-designated 503B pharmacies and US-based drug manufacturers. Note that 503B pharmacies are a relatively new type of pharmacy created by the Drug Quality and Security Act (HR3204) in 2013. They are licensed at both the state and federal (FDA) level. 503B pharmacies are permitted to manufacture sterile and non-sterile products that are found on the FDA Drug Shortage list.

Some smaller rural hospitals experienced sudden local outbreaks of COVID-19. Those rural facilities affiliated with larger network hospitals were able to, in some cases, acquire investigational products and commercial products in short supply via their network partners. Networked hospital pharmacies, via close communication and partnership with smaller rural hospitals, can thus help address the drug shortages and improve care for underserved communities. This practice is likely to continue as hospital networks expand, and as networks become more sophisticated at group-wide drug procurement and distribution [23].

For PPE, the modified federal and state requirements put into practice to conserve supplies most likely will not continue past the pandemic. Pharmacies will need to continue working with other hospital departments to identify and qualify new PPE vendors and maintain adequate supplies.

### Expanded Vaccination and Clinical Practice

The role of pharmacies in providing vaccination services has been growing steadily for more than 25 years. Pharmacies are increasingly recognized as “mass vaccinators” [24]. For instance, pharmacists administered influenza vaccine to nearly one-third of all adults who were vaccinated in 2018–2019 [16]. Access to vaccines via community and hospital pharmacies improves immunization rates among under-vaccinated population sectors, especially as pharmacies routinely offer expanded hours, convenient locations, and lower costs. Since people visit pharmacies for reasons that have nothing to do with health care, pharmacy-based vaccination may expand the pool of people who decide to get vaccinated. In addition, data support the conclusion that pharmacies provide vaccination services in a more cost-effective manner then physician offices and hospital clinics [25]. Pharmacies are also better positioned to access multiple vaccine suppliers and are better able to maintain a wide variety of vaccine products when compared with clinics and physician offices.

Although all 50 states now allow pharmacists to provide some level of vaccination services, state regulations have long placed limits on this scope of practice. With the COVID pandemic, most states and the federal government (PREP Act) [15-17] have significantly broadened the range of practice and now permit vaccination of both children and adults, and allow use of a wider range of vaccine types, including the COVID-19 vaccines. Pharmacy vaccination practice, by making vaccination more convenient, accessible, and affordable, will likely continue to expand, especially as it addresses a critical unmet public health need.

### Practices That Raise Concern

The risks associated with reuse of PPE by pharmacy staff remain unknown. The FDA and state guidance documents permitting reuse note that it is only a temporary adjustment made in response to the pandemic. A return to standard practice will likely occur as soon as PPE supplies return to pre-pandemic levels.

### Preparation for Future Public Health Challenges

Many of these pharmacy-related changes were initially driven by drug and PPE shortages, so measures to shore up supplies, and control use locally, can be implemented on an institutional basis as soon as a health challenge is recognized.

## Conclusion

The COVID-19 pandemic radically altered pharmacy practice, resulting in a dramatic increase in off-site work by pharmacists, loosening of restrictions on supplying medications to research participants on protocols, novel methods of interacting with contract research organizations and research study monitors, and expansion of pharmacies’ role in administering vaccines. In addition, it provided an opportunity to demonstrate the value of research pharmacy formulation and stability testing to speed the development of novel agents to combat the pandemic. At the same time, it stressed pharmacies, as drug shortages threatened patient care; PPE shortages threatened the health of pharmacists and their staff, and the ability to safely compound drugs; and lockdowns required searching for secure, alternative methods to deliver drugs to patients. And it demonstrated the value of pharmacy networks that can come to the aid of individual pharmacies that suffer a disproportionate impact from a pandemic. Collectively, this experience has provided an opportunity to examine many aspects of pharmacy practice afresh and assess which novel responses are likely to bring lasting value if adopted broadly and permanently, and which should only be kept in place until the emergency passes. Pharmacies at CTSA sites have played an extremely important role in this process and are well positioned to lead the ongoing assessment of current practices and the adoption of new ones.
